# Immobilization Approach as a Creative Strategy to Remove Reactive Dye Red 195 and Cu^2+^ Ions from Wastewater Using Environmentally Benign Geopolymer Cement

**DOI:** 10.3390/polym15071797

**Published:** 2023-04-05

**Authors:** Doaa A. Ahmed, Morsy A. El-Apasery, Shereen M. Ragai

**Affiliations:** 1 Chemistry Department, Faculty of Women for Arts, Science and Education, Ain Shams University, Cairo 11757, Egypt; 2 Dyeing, Printing and Textile Auxiliaries Department, Textile Research and Technology Institute, National Research Centre, 33 El Buhouth St., Cairo 12622, Egypt

**Keywords:** reactive red 195, Cu^2+^ ions, immobilization, geopolymer cement, slag, fly ash

## Abstract

Water is a resource that is essential to almost all phases of industrial and manufacturing operations globally. It is important to handle the wastewater generated professionally. The textile industry is one of the major global polluters, with textile producers responsible for one-fifth of all industrial water pollution worldwide. In contrast, heavy metal contamination has developed into a critical, expanding global environmental problem. Geopolymer is a cementitious constituent of amorphous aluminosilicates derived from natural or industrial wastes. It is produced using the polymerization of aluminosilicate raw ingredients in an alkaline atmosphere. The aim of this study is to evaluate the application of eco-friendly geopolymer cement in the immobilization technique for the treatment of wastewater including heavy metals and dyes. Geopolymer cement pastes were organized using slag and fly ash as an aluminosilicate source, (1:1) sodium silicate and sodium hydroxide 15 wt.% as an alkali activator in the presence of organic dye pollutant reactive red 195, and Cu^2+^ ions (700 ppm) at different hydration times for up to 28 days. The physicochemical and mechanical properties of the prepared geopolymer cement mixes were further examined in relation to reactive dye pollutant and Cu^2+^ ions. The hydration characteristic was examined using the compressive strength and % of total porosity tests, as well as FTIR and XRD studies. Our findings support the 100% immobilization of both Cu^2+^ ions and organic dye pollutants in prepared geopolymer pastes for up to 28 days of hydration. Additionally, adding both Cu^2+^ ions and dye pollutants to the prepared geopolymer paste improves its mechanical properties, which is also supported by FTIR data. XRD and FTIR studies showed that the Cu^2+^ ions and dying bath effluent addition have no influence on the kind of hydration products that are produced. On the other hand, the geopolymerization process is negatively impacted by the presence of Cu^2+^ ions alone in the geopolymer paste.

## 1. Introduction

Water pollution has become a major threat to humans, animals, and plants in recent years, leading to issues with decreased crop yields, diminished aquatic life, disturbed ecological balance, contaminated drinking water, and other detrimental effects [1]. There are many, different contaminants, including heavy metals, dyes, medicines, surfactants, pesticides and personal care items. Heavy metals are regarded as a high-risk contaminant that can endanger both human health and the environment. Inorganic elements with densities more than 5 g/cm^3^ are referred to be heavy metals. They are primarily divided into two groups, essential and nonessential heavy metals [1,2]. 

Heavy metals that are essential for numerous biological processes in living things, such as Cu, Zn, Fe, Co, and Mn, are often safe at low concentrations. Heavy metals that are not necessary, nonetheless, such as Pb, Cd, Cr, As, and Hg, are widely regarded as harmful even at low concentrations because of their high toxicity level and bioaccumulation feature in food chains [2]. There are numerous methods for removing heavy metals, dyes, and other pollutants from wastewater, each with their own benefits and drawbacks, including ion exchange, supercritical fluid extraction, adsorption [3], filtration, electrocoagulation, precipitation [4], microbiological systems, electrochemical processes [5], biological treatment [6], coagulation–flocculation, chemical precipitation, floatation, and reverse osmosis [7,8]. The above-mentioned traditional techniques’ applicability is constrained by their process effectiveness, environmental friendliness, and economic viability [9].

Adsorption is the method that is most frequently used to remove heavy metals, dyes, and other contaminants from sewage because of its straightforward procedure, effective regeneration, and reusability [10]. To remove heavy metals, dyes, and other contaminants from wastewater, several different adsorbents are utilized, including zeolites, activated carbon, resins, fly ash, chitosan, and others [11,12,13,14]. Due to its large surface area, stability, and longevity, activated carbon is the most widely utilized adsorbent for the removal of contaminants from wastewater [11]. The implementation of this technology for large-scale treatment of wastewater is constrained by its costly synthesis and challenging regeneration [15,16]. The need for highly effective, economical, and ecologically friendly adsorbents is growing as a result. Geopolymers might be used for this as a potential alternative.

An inorganic polymer known as a geopolymer is made up of SiO_4_ and AlO_4_ tetrahedrons that are joined by exchanging oxygen atoms [17]. They can usually be created by low-temperature polycondensation of silica- and alumina-rich raw materials’ natural and byproducts, such as metakaolin, blast furnace slag, coal fly ash, and red mud [17,18,19]. Geopolymers may be essentially described as a three-dimensional, amorphous, or semicrystalline aluminosilicate framework. It has been regarded as a workable substitute for Portland cement due to its superior mechanical characteristics, durability, low energy requirements, and low greenhouse gas emissions [17]. In addition, geopolymers have gained a lot of attention recently, particularly in wastewater treatment for the removal of various organic and inorganic pollutants including heavy metals and dyes [17,18,19,20,21,22,23,24,25,26,27], using a variety of methods, primarily adsorption as well as photodegradation, encapsulation, and immobilization [17,28,29,30,31,32]. 

As geopolymers offer so many promising attributes as a cement substitute material in terms of financial, environmental, and technological advantages, a sizable body of literature has been devoted to their potential for usage in the building sector. Geopolymers have increasingly garnered a lot of attention for the immobilization of harmful wastes in recent years [17]. For the Solidification/Stablization of hazardous pollutants, which are classified into heavy metals (cations and anions) and organic pollutants, geopolymers are currently the topic of extensive several studies [17,33,34,35,36,37,38,39,40,41,42]. Particularly geopolymers have the potential to offer extraordinarily long-term storage for the safe disposal of radioactive waste with high radioactivity [17]. The stabilization/solidification method is a unique technique that has recently been investigated for the removal of dye-contaminated water [34,36,38].

Our most recent research [20,22] indicates that the best decolorization of the various reactive dye effluents is achieved using a geopolymer made of fly ash and slag with a mole ratio of 90:10 wt.%. This study’s goal is to develop a novel, environmentally safe, practical technique for eliminating the color of the reactive red dye 195 remaining in the dyeing bath in combination with heavy metals, rather than just disposing of this hazardous waste without treatment. In our investigation, the solidification/stabilization technique was used to treat a mixture of wastes from organic dyes (reactive red 195 dye) and heavy metals (Cu^2+^ ions) by geopolymerization using cementitious materials. Three different geopolymer mixes based on slag incorporated with fly ash (SF-RD, SF-RD-Cu^2+^, and SF-Cu^2+^) were subjected to a solidification/stabilization test. The test was carried out for different hydration periods from 1 day to 28 days. Our results indicate that the eco-friendly slag/fly ash geopolymer paste has superior stabilization behavior for Cu^2+^ ions and reactive red 195 pollutants.

## 2. Materials and Methods

### 2.1. Materials

The raw materials and their chemical oxide composition used to prepare slag/fly-ash-based geopolymer (SF) are the same as in our previous studies [20,22,34,38]. GGBFS, or ground granulated blast furnace slag, was obtained from Egyptian Iron & Steel’s Helwan Company, Cairo, Egypt. Slag has a Blaine surface area per gram of 4700 × 50 cm^2^. Class F fly ash (FA) was provided by the Sika Chemical Company, located in Burg Al-Arab, Egypt. Sodium hydroxide was supplied by the EL-Goumhouria Chemical Company in Cairo, Egypt. The Silica Egypt Company in Alexandria, Egypt, provided industrial liquid sodium silicate (LSS), with SiO_2_/Na_2_O of 2.80. The chemical oxide composition for raw materials can be seen in Table 1. In addition, the copper chloride anhydrous used in this study was provided by Merck (Darmstadt-Germany) with 99% purity.

#### 2.1.1. Dyeing Procedures

##### **A**-Dyeing of Cotton Fabric

**Figure 1:**
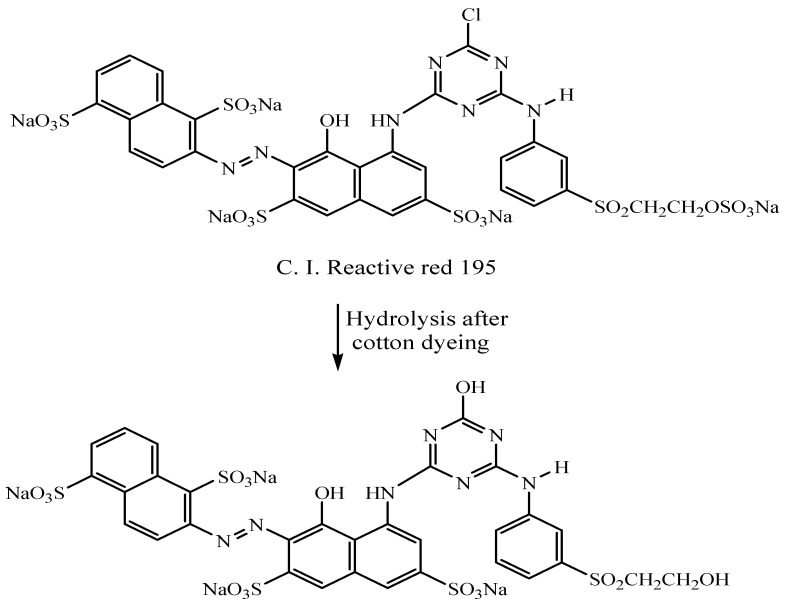
C.I. Reactive red 195 and hydrolyzed reactive red 195.

Amounts 60 g/L of sodium sulfate and then 20 g/L of sodium carbonate were applied to reactive red 195 dye (2% shade, 0.8 mg). Sodium sulfate was used to dye 40 mg of cotton fabrics for 30 min at 40 °C, followed by 60 min at 60 °C for fixation in sodium carbonate. The colored sample was thoroughly rinsed and then allowed to air-dry. Figure 1 illustrates the dyes’ structure.

##### **B**-Dyeing of Wool Fabrics

Wool fabric was dyed conventionally using conventional heating at temperatures of 80 °C for 60 min in a hydrolyzed reactive dye bath containing the remaining varied amounts of dye with a liquor ratio of 50:1 (Figure 1). The samples that had been dyed were rinsed in cold water and dried at room temperature. It is important to note that the dye concentration used in the dyeing of cotton or wool fibers was 15%, while the remaining dye proportion was 85%, or 0.68 mg/L. The color strengths of different dyed fabrics are illustrated in Table 2.

### 2.2. Methods

#### 2.2.1. Preparation of Geopolymer Cement Samples

Dry components of 90% slag (GGBFS) and 10% fly ash (FA) must be carefully combined to attain complete homogeneity when producing different geopolymer pastes. The various alkaline activator solutions (in the absence and presence of reactive red dye effluent and Cu^2+^ ions) were produced by mixing liquid sodium silicate and sodium hydroxide pellets at a specific ratio of 15:15 wt.% from solid content until an uniform gel was formed [43]. The concentration of the copper chloride solution used in our investigation was 700 ppm. Replace the mixing water added to prepare the alkaline activator with 100 mL from each of the dye effluent and Cu^2+^ ion solutions separately and with 100 mL from the combination of both. Geopolymer pastes were produced by combining dry constituents with appropriate alkaline activator solutions until they create a homogeneous paste. The water consistency of the geopolymer pastes was tested by standard Vicat equipment after complete mixing [44]. Table 3 provides the individual mixes’ constituents and the water/solid ratio that gave standard consistency. The mixtures were then set in 1-inch stainless steel molds (cubic-shaped molds). A thin-edged trowel was used to smooth the paste’s surface. Directly after casting, the pastes were cured at room temperature at 100% relative humidity for 24 h.

#### 2.2.2. Curing of the Slag-FA-Based Geopolymer Mixes

The cubes were taken out of the mold, and then six cubes from each geopolymer mix were curing for 1, 3, 7, 14, and 28 days in 100 mL distilled water, to test their leachability and mechanical properties. The cubes were taken out of their curing condition after each hydration interval, and the compressive strength and total porosity% data were checked.

#### 2.2.3. Physicochemical and Mechanical Tests of Prepared Geopolymer Cement Samples’

##### (A) Compressive Strength and Stopping of Hydration Tests

Using a set of three cubes, the compressive strength of each hardened paste was calculated. As the average of the three values, the results are given in kg/cm^2^. Compressive strength in this study was assessed using a manually operated compression testing machine (D550 control type, Milano-Italy). The stopping of hydration was performed on the crushed cubic specimens after the compressive strength test. The crushed samples were collected, mixed with a stopping mixture of alcohol and acetone (1:1) to stop further hydration, and dried at 50 °C for 24 h before being kept for further analysis (XRD, FTIR).

##### (B) Total Porosity Measurement

Total porosity examinations were carried out by weighing samples of dry pastes suspended in water and air, designated W_1_ and W_2_, for three separate cubes. Then, for around 24 h, these cubes were dried at 100 °C to calculate their weight in the atmosphere, W3. The total porosity percentage (P%) was calculated using the following Equation (1):

P% = [(W_1_ − W_3_)/ (W_1_ − W_2_)] ×100 (1)

##### (C) X-ray Diffraction Analysis (XRD)

The phase composition of the hydration products of various geopolymer mix samples was analyzed using X-ray diffraction analysis. Cu-K radiation with a wavelength of =1.5418 A and a pixel detector set to 40 kV and 40 mA was used for XRD analysis with a Ni-filtered diffractometer (Empyrean diffractometer, Holland).

##### (D) Fourier Transform Infrared Spectroscopy (FTIR)

The functional groups of the generated hydration products were identified using FTIR measurements. It was carried out on an infrared spectrophotometer using pellets of potassium bromide (KBr) (PerkinElmer 1430 infrared spectrophotometer, USA). Wave numbers in infrared spectra ranged from 400 to 4000 cm^−1^.

#### 2.2.4. Leaching Test for Curing Solutions after Different Hydration Periods

The concentration of copper ions in the leachate solution was evaluated using an atomic absorption spectrophotometer (Savant AA-GBC Scientific Equipment, Australia). Moreover, a spectrophotometer (V-670) was used to measure the amount of reactive red 195 dye effluent present in the leachates after each hydration time. 

In order to estimate the leaching percentage (% L), Equations (2) and (3) were applied [36]:

Leaching % = (C_L_/C_T_) × 100 (2)

where C_L_ is the concentration of the dye or copper ions leached out (mg/L) of the geopolymer mix cube, and C_T_ is the total concentration (mg/L) of the organic dye effluent or Cu^2+^ ions incorporated in the geopolymer mix [34].

Leaching % = (X/Original wt.) 100(3)

(X (grams in L) = Concentration (ppm) × 10^−3^, original weight of metal ion = wt. of cube × original concentration × total leachate volume)

Equation (4) [34,37] can be used to calculate how much copper ions and organic dye effluent are immobilized in the hardened geopolymer pastes

Immobilization = 100 − Leaching %(4)

#### 2.2.5. pH Measurements

An Adwa AD1000 pH meter (Szeged-Hungary) was used to measure the pH of leaching solutions across time (0, 1, 3, 7, 14, and 28 days) for each geopolymer paste.

## 3. Results and Discussion

### 3.1. Physicochemical and Mechanical Characteristics of Various Geopolymer Mixes

#### 3.1.1. Visual Appearance

**Figure 2:**
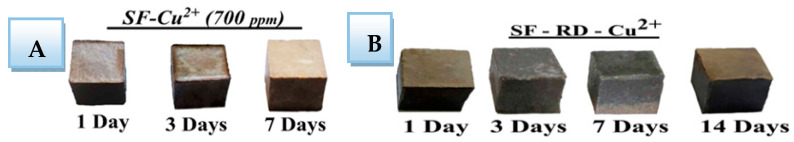
Visual observation of different slag-FA-based geopolymer mixes activated by NaOH and Na_2_SiO_3_ after different periods of water curing, (**A**) SF-Cu^2+^ and (**B**) SF-RD-Cu^2+^.

**Figure 3:**
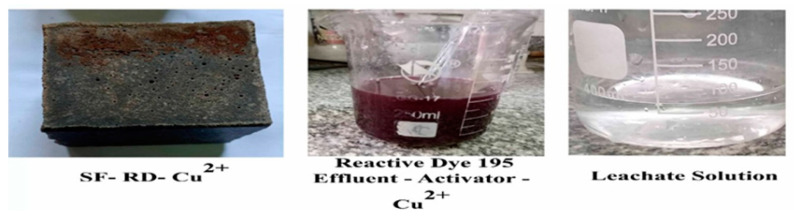
Solidification of a mixture from copper (II) ions and reactive red 195 dyeing bath effluent with slag-FA-based geopolymer mix after 7 days of curing condition.

Figure 2A,B show the physical observations for slag incorporated with FA geopolymer specimens (SF) in the presence of copper (II) ions and organic dye effluent curing for different hydration times. The results of a leaching test for copper (II) ions mixed with reactive red 195 dyeing bath effluent using an SF geopolymer mix after curing in water for 7 days are shown in Figure 3. The presence of dyeing bath effluent and copper (II) ions has an effect on the color of the geopolymer cubes, as can be seen in Figure 2 and Figure 3.

#### 3.1.2. Compressive Strength

**Figure 4:**
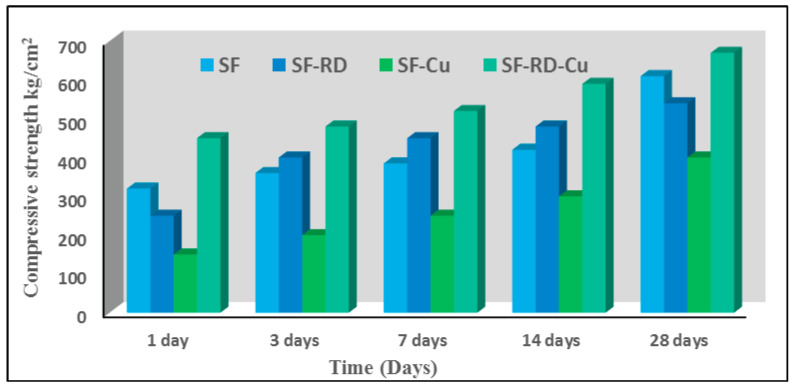
Compressive strength of different geopolymer cement mixes after curing at different hydration ages.

Figure 4 illustrates the compressive strength properties of hardened slag/fly-ash-based geopolymer mixes (SF, SF-D, SF-RD-Cu^2+^, and SF-Cu^2+^) cured in water for variable hydration durations of 1, 3, 7, 14, and 28 days. The compressive strength values, of all geopolymer mixes as shown in Figure 4, rise with increasing curing time. This is explained by the fact that the presence of a high alkali medium increases the dissolution rate of slag and fly ash, providing the system with silicate and aluminate species, which complete the geopolymerization process. In contrast, this led to an increased hydrating rate and the production of additional hydration products, such as CSH, CASH, and N-(C)ASH gel, that precipitated in the open pores, enhancing the compressive strength [17]. Compared with other mixes, the values for compressive strength for SF-RD-Cu^2+^ mixes exhibited the highest values, which might be explained by the incorporation of dyeing bath effluent and copper (II) ions into the slag-based geopolymer matrix, which reduced the pore size. Figure 4 also reveals that the compressive strength values for the SF-Cu^2+^ mix were the lowest, which, in accordance with the most recent study [35,37], may be explained by the retardation effect of heavy-metal-ion-induced hydration processes.

#### 3.1.3. Total Porosity (P%)

**Figure 5:**
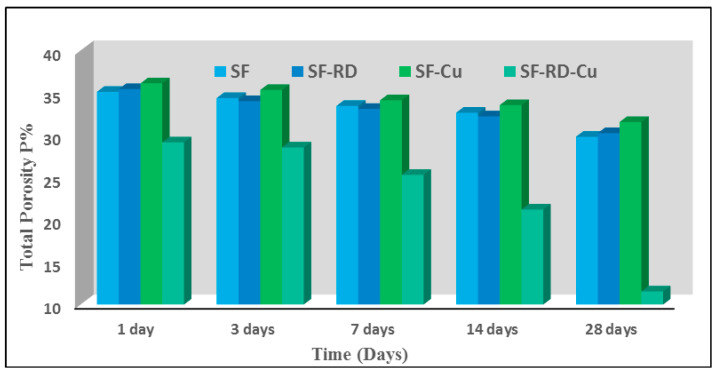
Total porosity% of different geopolymer cement mixes after curing in different hydration ages.

The total porosity of specimens of hardened SF, SF-Cu^2+^, SF-RD, and SF-RD-Cu^2+^ that were cured for 1, 3, 7, 14, and 28 days is shown in Figure 5. For all geopolymer mixes, it has been demonstrated that total porosity% decreases with curing time. These findings provide an indication as to how the polymerization process is progressing and how much more amorphous silicate gel is forming inside the matrix [17,34,38]. In contrast with other geopolymer mixes, the addition of both copper ions and reactive red 95 dyeing bath effluent in the geopolymer mix SF-RD-Cu^2+^ reduces the total porosity% values at all hydration time (Figure 5). This could be because the alkaline activator’s hydroxyl ions react with copper (II) ions to form Cu(OH)_2_, which precipitates inside the geopolymer matrix and reduces the size of the pores. Additionally, the presence of dyeing bath effluent accelerates the geopolymerization process, which may be attributed to the change in the dye structure after fiber treatment into hydroxyl form. This may cause the pH of the medium to rise, accelerating the formation of more hydration gel products and filling open pores. The geopolymer mix SF-Cu^2+^, on the other hand, demonstrated the highest values of total porosity% after all hydration periods. It can be attributed to the consumption of hydroxyl ions by the formation of Cu(OH)_2_, which resulted in a delay in the geopolymerization process and the formation of less hydrated products, leading to an increase in the size of pores [17,35].

#### 3.1.4. Fourier Transform Infrared Spectroscopy (FTIR)

**Figure 6:**
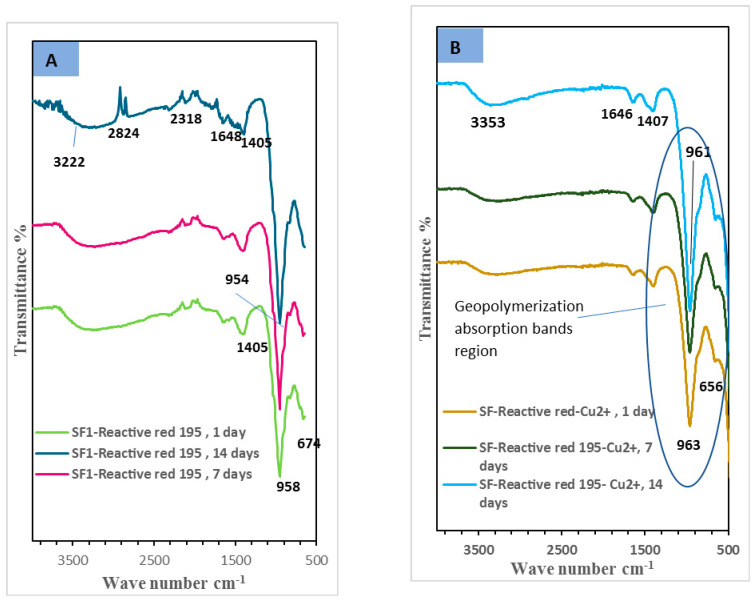
FTIR spectra of different geopolymer mixes cured in water for different hydration ages, (**A**) SF-RD, (**B**) SF-RD-Cu^2+^.

**Figure 7:**
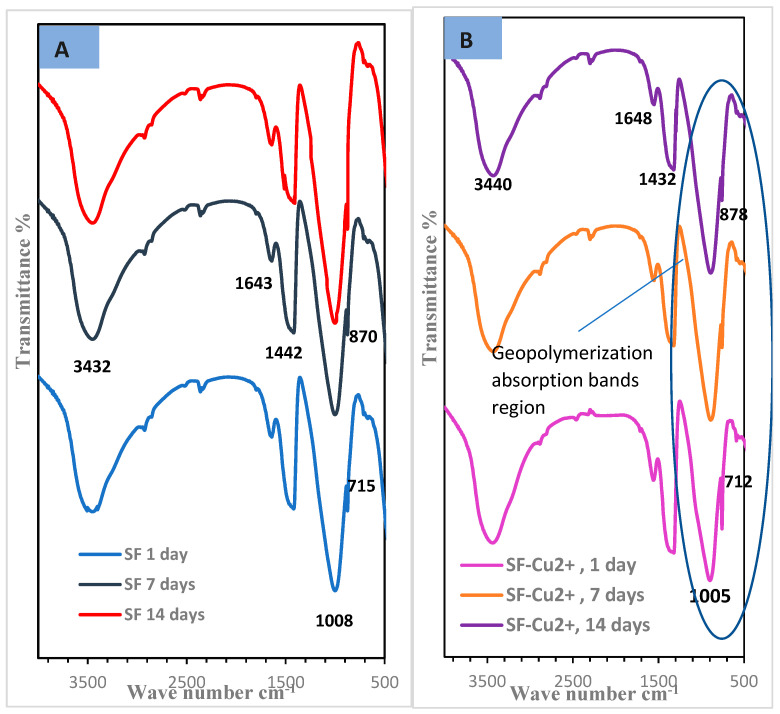
FTIR spectra of different geopolymer mixes cured in water for different hydration ages, (**A**) SF, (**B**) SF-RD-Cu^2+^.

Figure 6 illustrates the FTIR of slag/fly-ash-based geopolymer mixes in the presence of reactive red 195 dye effluent as well as both dyeing bath effluent and Cu^2+^ ions (SF-RD and SF-RD-Cu^2+^) after various hydration periods. Figure 7, in contrast, shows the IR bands related to SF and SF-Cu^2+^ geopolymer composites curing in H_2_O for different hydration ages. The FTIR spectra, for all geopolymer mixes, exhibit absorption bands related to both the stretching and bending vibration modes of the O-H group and the H-O-H group, respectively, in the ranges of 3440–3218 cm^−1^ and 1643–1648 cm^−1^. This demonstrates the dissolution and condensation of slag and fly ash species by applying an alkaline activator solution, resulting in the creation of excessive amounts of hydration products inside the geopolymer matrix [45,46,47]. The spectra also show the appearance of two absorption bands related to the CO group, which is formed during the carbonation process of calcium hydroxide; these bands are around 1405–1445 cm^−1^, which are related to the stretching vibration of the C-O bond, and 870–875 cm^−1^, which are related to the out-of-plane bending vibration of CO_2_. Figure 3 shows that applying reactive red 195 dyeing bath effluent, either alone or in combination with copper ions, to the geopolymer matrix reduced the carbonation process. In contrast, we recognize from Figure 7B that the carbonation process is enhanced by the addition of copper (II) ions alone to the geopolymer paste by intensifying the CO-group-related absorption bands. This could be explained by copper ions reducing the geopolymerization process [35]. We also noticed that the major and strong absorption band at around 1008 cm^−1^ that corresponds to the asymmetric stretching vibrations of Si-O-T (where T = Si or Al) seen in the SF mix’s IR spectrum (Figure 7A) shifted to a lower wavenumber at around 980–954 cm^−1^ for the geopolymer mixes SF-RD and SF-RD-Cu^2+^ (Figure 6). The geopolymerization process and the development of amorphous aluminosilicate gels (CSH and N-(C)-A-S-H) in geopolymer binders can be regarded as explanations of the band shift [48,49]. These findings demonstrate the positive benefits of reactive red 195 dyeing bath effluent alone or in combination with copper (II) ions on the geopolymerization process and production of additional hydration products. As we notice from Figure 7, the decrease in the intensity of the main absorption band related to ν_as_ of Si-O-T (at 1005 cm^−1^) in the case of the SF-Cu^2+^ mix is further evidence of the geopolymerization retardation effect of copper ions. 

#### 3.1.5. X-ray Diffraction Analysis (XRD)

**Figure 8:**
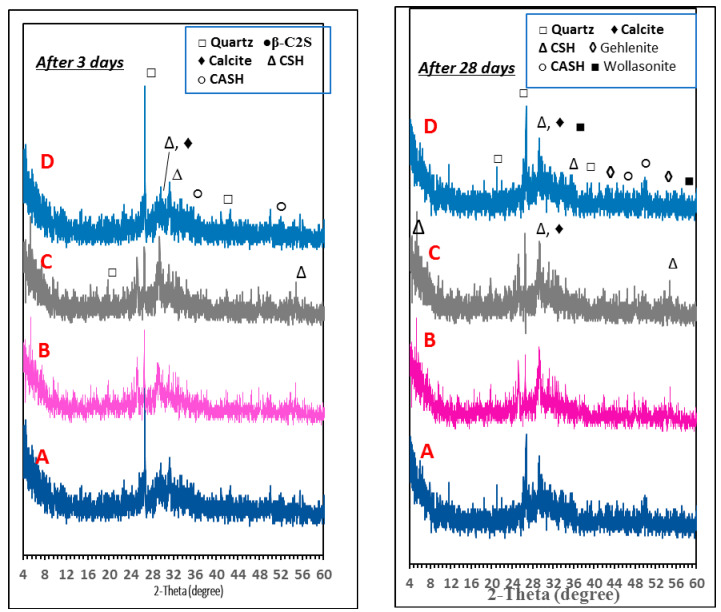
X-ray diffraction pattern of different geopolymer mixes under water curing for 7 and 28 days: (**A**) SF, (**B**) SF-RD, (**C**) SF-RD-Cu^2+^, (**D**) SF-Cu^2+^.

The XRD patterns of the geopolymer mixes (SF, SF-dye, SF-Cu^2+^, and SF-RD-Cu^2+^) after 3 and 28 days of hydration curing in water are shown in Figure 8. As a result of the complete dissolution of slag and fly ash species by alkaline activation and the development of an amorphous geopolymer matrix, all geopolymer samples showed a huge and broad hump between 20° and 40° 2θ in their XRD patterns [50]. The major hydration product of the geopolymerization process was identified as the crystalline phase, (CSH) with basal reflections at d = 3.03 and 2.786 Aº, respectively. Additionally, Figure 8 illustrates a significant quartz peak at d = 3.34, 4.25, 2.45, and 1.81 Aº, which could be caused by unreacted silica from the raw materials. The release of free silica that has been partially substituted with aluminum (Al) atoms or that has interacted with hydrated calcium oxide suggests the creation of a new crystalline phase, such as (CASH) type gel (d = 1.97 Aº). Figure 8 demonstrates that the nature of the hydration product generated in geopolymer pastes is unaffected by the presence of reactive red 195 dyeing bath effluent and copper ions. According to the XRD patterns, the presence of copper ions alone in the geopolymer mix has caused a drop in the intensity of the characteristic CSH peaks. As shown in Figure 8, however, reactive red 195 dyeing bath effluent improves hydration by increasing the intensity of CSH peaks and decreasing the intensity of the peak related to quartz. The development of a well-resolved crystalline peak associated with gehlenite (Ca_2_Al_2_SiO_7_) (d-values = 3.066, 2.84, 2.74, 1.91, 1.81 Aº) and wollastonite (CaSiO_3_) (d = 2.97, 2.63, 1.86 Aº) was observed as a result of paste sintering (denser microstructure) [35]. These are dehydrated forms of phases that provide strength, such as calcium silicate hydrate (CSH) and calcium aluminum silicate hydrate (CASH).

### 3.2. Leaching of Reactive Red 195 Dye Effluent and Copper (II) Ions from Slag-FA-Based Geopolymer Mixes

#### 3.2.1. Optical Absorption Spectra for Reactive Red 195 Dying Bath Effluent in Leachate Solutions

**Figure 9:**
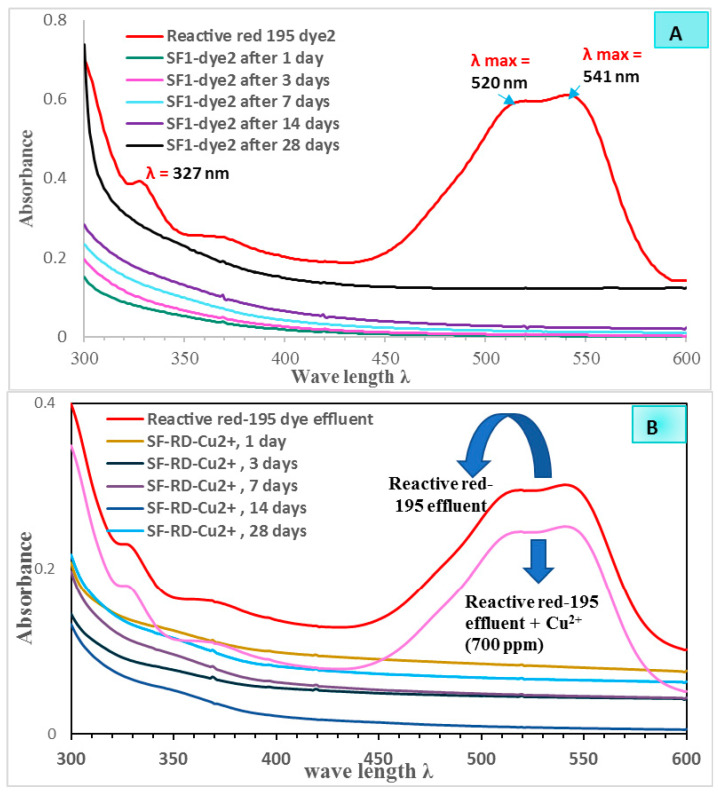
UV–VIS absorption spectra of reactive red 195 dyeing bath effluent and different leachate solutions for: (**A**) *SF-RD* geopolymer mix and (**B**) *SF-RD-Cu^2+^*, after different curing periods.

Figure 9 demonstrates the immobilization behavior of different geopolymer mixes (SF-dye, SF-D-Cu^2+^) against reactive red 195 dying bath effluent (0.68 mg/L) as determined by UV–VIS absorbance spectra of leachate solutions following various curing times of 1, 3, 7, 14, and 28 days. As shown in Figure 9, the reactive red 195 dye’s UV–VIS absorption band spectra were recorded a maximum at various wavelengths of 327, 520, and 541 nm. As we notice from Figure 9A,B, the absorption measurements of the leachate solutions for the different prepared geopolymer mix cubes SF-RD and SF-RD-Cu^2+^ cured from 1 day to 28 days in H_2_O showed a complete disappearance of the absorption band maxima related to the reactive red 195 dye (at 321, 520, and 541 nm). This could be explained by the better adsorption capabilities of the geopolymer matrix and its hydration products (CSH and CASH), which make it easier to become removed of wastes, such heavy metals and organic dyes affluent, as the results showed in a previous studies [34,36,38]. Table 4 shows the calculated values of leaching % and immobilization of reactive red 195 dyeing bath effluent in different leachate solutions after various curing periods. These data show that the color of reactive red 195 dyeing bath effluent is eliminated by stabilizing and hardening inside the geopolymer matrix.

#### 3.2.2. Determination of Copper (II) Ions in Geopolymer Mix Leachate Solutions

Using the atomic absorbance spectroscopy measurement of leachate solutions after different curing durations from 1 day to 28 days, the effectiveness of immobilizing of copper ions (700 ppm) in the absence and presence of effluents of reactive red 195 dye inside the slag-FA-based geopolymer matrix was investigated. The results of the leaching % of the several geopolymer mixes, including SF-Cu^2+^ and SF-RD-Cu^2+^, are summarized in Table 5. After 28 days of hydration, it is seen that the leaching % of copper (II) ions in the case of the geopolymer mixes SF-RD-Cu^2+^ equals zero. In the case of the SF-Cu^2+^ geopolymer mix, however, the leaching% values of copper ions are extremely low, and the immobilization values are close to 100. It is explained by the geopolymer paste’s remarkable efficiency in immobilizing copper ions, which fully solidify inside the geopolymer matrix over a period of the 28 days, as well as the beneficial effects of reactive red 195 dyeing bath effluent on the copper ion immobilization process. This highlights the slag/fly-ash-based geopolymer’s exceptional effectiveness in trapping copper ions and reactive red 195 dyeing bath effluent and converting harmful slag and fly ash into user- and environmentally building materials. 

#### 3.2.3. Leachate Solution pH Measurements as a Function of Contact Time with Various Geopolymer Mixtures

**Figure 10:**
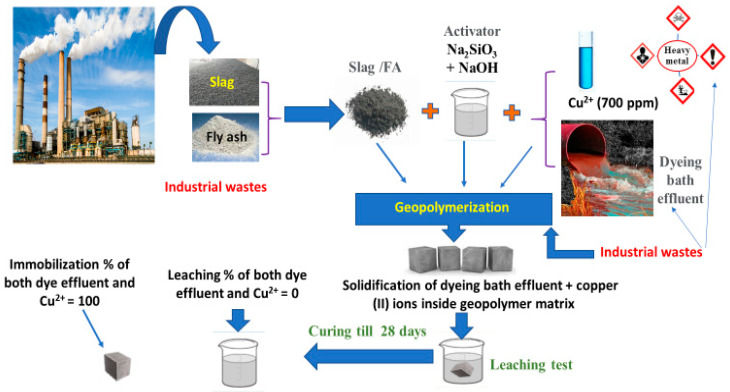
Schematic of environmentally friendly disposal of industrial wastes by using a geopolymerization strategy.

According to Table 6, several geopolymer mix cubes containing reactive red 195 dyeing bath effluent and copper (II) (700 ppm) (SF-Cu^2+^, SF-dye, and SF-D-Cu^2+^) were tested to see how the pH of the leachate solutions changed with contact time (0, 3 h, 1, 3, 7, and 14 days). The pH values for several geopolymer mix leachate solutions increased throughout the duration of 28 days of hydration. These high levels of alkalinity are induced by the continuous hydration of the geopolymer paste over time and the release of alkaline species into leaching solutions [34,36,38]. We have shown in Figure 10 a schematic diagram of the environmentally friendly disposal of industrial wastes through the use of the geopolymerization process.

#### 3.2.4. Comparison between Adsorption and Immobilization Efficiency of Slag-Based Geopolymer Incorporated with Fly Ash

The adsorption of reactive red 195 dyeing bath effluent by an SF geopolymer mix was evaluated according to our last study [22]. The results indicate that the maximum removal efficiency (%) of the reactive red 195 dyeing bath effluent in the geopolymer cement mix SF was equal to **91.3** for a dye concentration (5 ppm) at pH 3 after 1 h [22]. Table 7 summarizes the data from our current and previous studies to demonstrate the differences between the two approaches used to remove the reactive red 195 dyeing bath effluent. The findings suggest that the solidification approach offers an effective method for disposing of dangerous and toxic contaminants.

## 4. Conclusions

In our present work, the immobilization behavior of different geopolymer cements constructed from slag incorporated with fly ash towards both copper ions (700 ppm) and reactive red 195 dying bath effluent was studied. The hydration characteristics of the different prepared geopolymer mixes in the presence of Cu^2+^ ions (700 ppm), in addition to the reactive red 195 dying bath effluent, were also analyzed for different hydration ages from 1 day to 28 days. Our data indicate that:

- The developed eco-friendly geopolymer pastes eliminate copper ions and dyeing bath effluent.
- The leaching test indicates complete removal of both pollutants by solidification inside the geopolymer matrix for different hydration ages and till 28 days.
- The geopolymer mix’s compressive strength and porosity values are enhanced by the addition of reactive red 195 dyeing bath effluent and Cu^2+^ ions.
- The nature of the hydration products formed is unchanged by the addition of Cu^2+^ ions and dying bath effluent, according to XRD and FTIR analysis.
- The presence of dyeing bath effluent inside the geopolymer matrix accelerates the geopolymerization process, which was proven by FTIR and XRD analysis.
- The presence of copper ions alone in the geopolymer matrix has a retardation effect on the geopolymerization process.
- The high alkalinity of curing solutions especially at the first hydration age is due to the progress of geopolymerization.
- The solidification approach seems to be a more effective technique to remove hazardous and toxic materials when compared with the adsorption method.

Our findings suggest that a geopolymer binder derived from industrial waste based on 90% slag and 10% fly ash can be used to remove organic dye pollutants simultaneously with copper ions with 100% removal efficiency using solidification method.

## Figures and Tables

**Table 1:** Chemical composition of starting materials by XRF, mass %.

	Chemical Composition %										
	* **SiO_2_** *	* **Fe_2_O_3_** *	* **CaO** *	* **Al_2_O_3_** *	* **Cl^−^** *	* **MgO** *	* **SO_3_** *	* **Na_2_O** *	* **K_2_O** *	* **L.O.I** *	* **H_2_O** *
**Slag**	32.86	1.14	42.56	7.02	0	11.58	2.50	0.29	0.15	0.93	0
**FA**	63.10	5.40	2.33	26.54	0.85	0.52	0	0	0.09	0	0
**LSS**	32.8	0	0	0	0	0	0	11.7	0	0	55.5

**Table 2:** Color strengths of fabrics dyed with reactive red 195.

Fabrics	Color Strength K/S	L *	a *	b *
**Cotton**	23.44	61.09	49.85	−6.17
**Wool**	20.69	59.14	48.65	−6.02

**Table 3:** Mix composition of the examined mixture and liquid/solid (L/S) ratio.

Mixes Name	Slag	FA	Reactive Red Dye Effluent mL	Cu^2+^ (700 ppm) mL	NaOH wt.%	Na_2_SiO_3_ wt.%	L/S Ratio
SF	90	10	------	------	15	15	0.47
SF-Cu^2+^	90	10	------	100	15	15	0.35
SF-RD-Cu^2+^	90	10	100		15	15	0.48
SF-RD	90	10	100	-------	15	15	0.42

**Table 4:** Leaching % and immobilization values of reactive red 195 dyeing bath effluent in leachate solutions of different geopolymer mixes after different curing times.

Time (Days)	Leaching %		Immobilization (100-Leaching %)	
	*SF-RD*	*SF-RD-Cu^2+^*	*SF-RD*	*SF-RD-Cu^2+^*
**1**	0	0	100	100
**3**	0	0	100	100
**7**	0	0	100	100
**14**	0	0	100	100
**28**	0	0	100	100

**Table 5:** Leaching % and immobilization values of copper (II) ions (700 ppm) in leachate solutions of different geopolymer mixes after different curing times.

Time (Days)	Leaching %		Immobilization (100-Leaching %)	
	SF-Cu^2+^	SF-RD-Cu^2+^	SF-Cu^2+^	SF-RD-Cu^2+^
**1**	0.00000271	0	99.999997	100
**3**	0.00000469	0	99.999995	100
**7**	0.00000426	0	99.999995	100
**14**	0.0000610	0	99.999993	100
**28**	0.0000945	0	99.999905	100

**Table 6:** pH’s values of leaching solutions of different mixes at different hydration ages.

Geopolymer Mixes	pH						
	0	3 h	1 Day	3 Days	7 Days	14 Days	28 Days
**SF-RD**	7.22	12.63	13.25	13.28	13.38	13.40	13.70
**SF-RD-Cu^2+^**	7.22	12.37	13.34	13.40	13.42	13.43	13.45
**SF-Cu^2+^**	7.22	12.62	13.40	13.43	13.45	13.45	13.44

**Table 7:** Comparison between immobilization and adsorption removal % values for different hazardous materials by using slag/fly-ash-based geopolymer (SF).

Geopolymer Mix/Dye or Heavy Metals	Reactive Red 195 Dyeing Bath Effluent		Copper (II) Ions (700 ppm)
	Adsorption Removal Efficiency % (after 1 h)	Immobilization Removal % (after 28 Days)	Immobilization Removal % after 28 Days
**SF + RD**	91.3 ***Elapasery et al.*** [22]	100 **(*present study*)**	**--------**
**SF + RD + Cu^2+^ (700 ppm)**	**-------**	100 **(*present study*)**	100 **(*present study*)**
**SF + Cu^2+^ (700 ppm)**	**------------**	**-------------**	99.999905 **(*present study*)**

## Data Availability

Not applicable.

## References

[1] Siyal A.A., Shamsuddin M.R., Khan M.I., Rabat N.E., Zulfiqar M., Man Z., Siame J., Azizli K.A. (2018). A review on geopolymers as emerging materials for the adsorption of heavy metals and dyes. J. Environ. Manag..

[2] Tan T.H., Mo K.H., Ling T.C., Lai S.H. (2020). Current development of geopolymer as alternative adsorbent for heavy metal removal. Environ. Technol. Innov..

[3] Lesmana S.O., Febriana N., Soetaredjo F.E., Sunarso J., Ismadji S. (2009). Studies on potential applications of biomass for the separation of heavy metals from water and wastewater. Biochem. Eng. J..

[4] Boamah P.O., Huang Y., Hua M., Zhang Q., Wu J., Onumah J., Sam-Amoah L.K., Boamah P.O. (2015). Sorption of heavy metal ions onto carboxylate chitosan derivatives—A mini-review. Ecotoxicol. Environ. Saf..

[5] Ahmed M.J.K., Ahmaruzzaman M.J. (2016). A review on potential usage of industrial waste materials for binding heavy metal ions from aqueous solutions. J. Water Process Eng..

[6] Guieysse B., Norvill Z.N. (2014). Sequential chemical–biological processes for the treatment of industrial wastewaters: Review of recent progresses and critical assessment. J. Hazard. Mater..

[7] O’Connell D.W., Birkinshaw C., O’Dwyer T.F. (2008). Heavy metal adsorbents prepared from the modification of cellulose: A review. Bioresour. Technol..

[8] Fu F., Wang Q. (2011). Removal of heavy metal ions from wastewaters: A review. J. Environ. Manag..

[9] Blackburn R.S. (2004). Natural polysaccharides and their interactions with dye molecules: Applications in effluent treatment. Environ. Sci. Technol..

[10] Acisli O., Acar I., Khataee A. (2020). Preparation of a fly ash-based geopolymer for removal of a cationic dye: Isothermal, kinetic and thermodynamic studies. J. Ind. Eng. Chem..

[11] Badawi M.A., Negm N.A., Abou Kana M.T.H., Hefni H.H., Moneem M.A. (2017). “Adsorption of aluminum and lead from wastewater by chitosan-tannic acid modified biopolymers: Isotherms, kinetics, thermodynamics and process mechanism. Int. J. Biol. Macromol..

[12] Li L., Zou J., Han Y., Liao Z., Lu P., Nezamzadeh-Ejhieh A., Liu J., Peng Y. (2022). Recent advances in Al(iii)/In(iii)-based MOFs for the detection of pollutants. New J. Chem..

[13] Zheng M., Chen J., Zhang L., Cheng Y., Lu C., Liu Y., Singh A., Trivedi M., Kumar A., Liu J. (2022). Metal organic frameworks as efficient adsorbents for drugs from wastewater. Mater. Today Commum..

[14] Zhong Y., Chen C., Liu S., Lu C., Liu D., Pan Y., Sakiyama H., Muddassir M., Liu J. (2021). A new magnetic adsorbent of eggshell-zeolitic imidazolate framework for highly efficient removal of norfloxacin. Dalton Trans..

[15] Holkar C.R., Jadhav A.J., Pinjari D.V., Mahamuni N.M., Pandit A.B. (2016). A critical review on textile wastewater treatments: Possible approaches. J. Environ. Manag..

[16] Albayati T.M., Alwan G.M., Mahdy O.S. (2017). High performance methyl orange capture on magnetic nanoporous MCM-41 prepared by incipient wetness impregnation method. Korean J. Chem. Eng..

[17] Tian Q., Bai Y., Pan Y., Chen C., Yao S., Sasaki K., Haijun, Zhang H. (2022). Application of Geopolymer in Stabilization/Solidification of Hazardous Pollutants: A Review. Molecules.

[18] Acisli O., Acar I., Khataee A. (2022). “Preparation of a surface modified fly ash-based geopolymer for removal of an anionic dye: Parameters and adsorption mechanism. Chemosphere.

[19] El Alouani M., Saufi H., Moutaoukil G., Alehyen S., Nematollahi B., Belmaghraoui W., Taibi M.H. (2021). Application of geopolymers for treatment of water contaminated with organic and inorganic pollutants: State-of-the-art review. J. Environ. Chem. Eng..

[20] Elapasery M.A., Ahmed D.A., Aly A.A. (2022). Decolorization of Reactive Dyes, Part II: Eco-Friendly Approach of Reactive Dye Effluents Decolorization Using Geopolymer Cement Based on Slag. Egypt. J. Chem..

[21] Elapasery M.A., Ahmed D.A., Aly A.A. (2022). Decolorization of Reactive Dyes, Part III: Eco-Friendly Approach of Reactive Dye Effluents Decolorization Using Geopolymer Cement Based on Metakaolin. Egypt. J. Chem..

[22] Elapasery M.A., Ahmed D.A., Aly A.A. (2022). Decolorization of Reactive Dyes, Part IV: Eco-Friendly Approach of Reactive Red 195 Dye Effluents Decolorization Using Geopolymer Cement Based on Slag. Egypt. J. Chem..

[23] Elapasery M.A., Ahmed D.A., Aly A.A. (2022). Decolorization of Reactive Dyes, Part V: Eco-Friendly Approach of Reactive Red 195 Dye Effluents Decolorization Using Geopolymer Cement Based on Metakaolin. Egypt. J. Chem..

[24] Ahmed S.M., Aly A.A., El-Apasery M.A., Ragai S. (2022). M Decolorization of Reactive Dyes, Part VI: Eco-Friendly Approach of Reactive Dye Effluents Decolorization Using Geopolymer Cement Based on Metakaolin backed by slag. Egypt. J. Chem..

[25] Ahmed S.M., Aly A.A., El-Apasery M.A., Ragai S. (2022). M Decolorization of Reactive Dyes, Part VII: Eco-Friendly Approach of Reactive Dye Effluents Decolorization Using Geopolymer Cement Based on Metakaolin-Slag mixes. Egypt. J. Chem..

[26] Ahmed S.M., Aly A.A., El-Apasery M.A., Ragai S. (2023). M Decolorization of Reactive Dyes, Part VIII: Eco-Friendly Approach of Reactive Red 195 Dye Effluents Decolorization Using Geopolymer Cement Based on Metakaolin backed by slag. Egypt. J. Chem..

[27] Ahmed S.M., Aly A.A., El-Apasery M.A., Ragai S. (2023). M Decolorization of Reactive Dyes, Part IX: Eco-Friendly Approach of Reactive Red 195 Dye Effluents Decolorization Using Geopolymer Cement Based on Metakaolin-Slag Mixes. Egypt. J. Chem..

[28] Li X., Bai C., Qiao Y., Wang X., Yang K., Colombo P. (2022). Preparation, properties and applications of fly ash-based porous geopolymers: A review. J. Clean. Prod..

[29] Panda L., Jena S.K., Rath S.S., Misra P.K. (2020). Heavy metal removal from water by adsorption using a low-cost geopolymer. Environ. Sci. Pollut. Res..

[30] Xu F., Gu G., Zhang W., Wang H., Huang X., Zhu J. (2018). Pore structure analysis and properties evaluations of fly ash-based geopolymer foams by chemical foaming method. Ceram. Int..

[31] Zhang P., Gao Z., Wang J., Guo J., Hu S., Ling Y. (2020). “Properties of fresh and hardened fly ash/slag-based geopolymer concrete: A review. J. Clean. Prod..

[32] Ariffin N., Abdullah M.M.A., Zaino R.R.M., Murshed M.F. (2017). Geopolymer as an adsorbent of heavy metal: A review. AIP Conf. Proc..

[33] Liu J., Zha F., Xu L., Yang C., Chu C., Tan X. (2018). Effect of chloride attack on strength and leaching properties of solidified/stabilized heavy metal contaminated soils. Eng. Geol..

[34] Elapasery M.A., Ahmed D.A. (2022). A Sustainable Approach for Immobilization Dyeing bath Effluents of Reactive Yellow 145 by using Different Types of Eco-Friendly Geopolymer Cement. Egypt. J. Chem..

[35] Ahmed D.A., Abdallah S.H., Ragai S.M. (2023). Hydration characteristic and leaching behavior of different mixes of slag based -geopolymer cement in presence of heavy metals. Egypt. J. Chem..

[36] Al-Mashaqbeha A., El-Eswed B., Banatc R., Khalilia F. (2018). Immobilization of organic dyes in geopolymeric cementing material. Environ. Nanotechnol. Monit. Manag..

[37] Helall S., Ahmed D.A., Ragei S.M. (2021). Effect of Pb^+2^ ion on physico- chemical properties of fly ash -slag geopolymer pastes. J. Sci. Res. Sci..

[38] Elapasery M.A., Ahmed D.A. An Innovative and Promising Facile Route to Remove Heavy Metals and Dyes using Environmentally Friendly Slag-based Geopolymer Cement. Egypt. J. Chem..

[39] Al-Harahsheh M.S., Alzboon K.K., Al-Makhadmeh L., Hararah M., Mahasneh M. (2015). Fly ash based geopolymer for heavy metal removal: A case study on copper removal. J. Environ. Chem. Eng..

[40] Darmayanti L., Notodarmodjo S., Damanhur E., Mukti R. (2018). Removal of Copper (II) Ions in Aqueous Solutions by Sorption onto Alkali Activated Fly Ash. MATEC Web Conf..

[41] Tan T.H., Mo K.H., Lai S.H., Ling T.C. (2022). Investigation on the copper ion removal potential of a facile-fabricated foamed geopolymer sphere for wastewater remediation. Clean. Mater..

[42] Xu J., Li M., Zhao D., Zhong G., Sun Y., Hu X., Sun J., Li X., Zhu W., Li M. (2022). Research and Application Progress of Geopolymers in Adsorption: A Review. Nanomaterials.

[43] Selim F., Ahmed D.A., Kishar E.A. (2021). Effect of Alkali Concentration on Physico-Chemical and Mechanical Properties of Slag Based Geopolymer Cement. J. Sci. Res. Sci..

[44] (2016). Standard Test Method for Normal Consistency of Hydraulic Cement.

[45] Khater H. (2013). Effect of cement kiln dust on geopolymer composition and its resistance to sulfate attack. Green Mater..

[46] Khater H., Ezzat M., El Nagar A. (2016). Engineering of Low Cost Geopolymer Building Bricks Applied For Various Construction Purposes. Int. J. Civ. Eng. Technol..

[47] Riyap H.I., Bewa C.N., Banenzoué C., Tchakouté H.K., Rüscher C.H., Kamseu E., Bignozzi M.C., Leonelli C. (2019). Microstructure and mechanical, physical and structural properties of sustainable lightweight metakaolin-based geopolymer cements and mortars employing rice husk. J. Asian Ceram. Soc..

[48] Criado M., Fernández-Jiménez A., Palomo A. (2007). Alkali activation of fly ash: Effect of the SiO_2_/Na_2_O ratio: Part I: FTIR study. Microporous Mesoporous Mater..

[49] Yang Z., Mocadlo R., Zhao M., Sisson R.D., Tao M., Liang J. (2019). Preparation of a geopolymer from red mud slurry and class F fly ash and its behavior at elevated temperatures. Constr. Build. Mater..

[50] Kassem N., Ahmed D., Kishar E. (2021). Effect of Elevated Temperatures on The Performance of Metakaolin Geopolymer Pastes Incorporated by Cement Kiln Dust. Egypt. J. Chem..

